# Shift and Mean Algorithm for Functional Imaging with High Spatio-Temporal Resolution

**DOI:** 10.3389/fncel.2015.00446

**Published:** 2015-11-17

**Authors:** Sylvain Rama

**Affiliations:** ^1^INSERM, UMR_S 1072Marseille, France; ^2^Unité de Neurobiologie des canaux Ioniques et de la Synapse (UNIS)Marseille, France; ^3^Department is UNIS, Unité de Neurobiologie des canaux Ioniques et de la Synapse, Aix-Marseille UniversityMarseille, France

**Keywords:** high-resolution, shift and mean, voltage sensitive dye imaging, calcium sensitive dye imaging, electrophysiology

## Abstract

Understanding neuronal physiology requires to record electrical activity in many small and remote compartments such as dendrites, axon or dendritic spines. To do so, electrophysiology has long been the tool of choice, as it allows recording very subtle and fast changes in electrical activity. However, electrophysiological measurements are mostly limited to large neuronal compartments such as the neuronal soma. To overcome these limitations, optical methods have been developed, allowing the monitoring of changes in fluorescence of fluorescent reporter dyes inserted into the neuron, with a spatial resolution theoretically only limited by the dye wavelength and optical devices. However, the temporal and spatial resolutive power of functional fluorescence imaging of live neurons is often limited by a necessary trade-off between image resolution, signal to noise ratio (SNR) and speed of acquisition. Here, I propose to use a Super-Resolution Shift and Mean (S&M) algorithm previously used in image computing to improve the SNR, time sampling and spatial resolution of acquired fluorescent signals. I demonstrate the benefits of this methodology using two examples: voltage imaging of action potentials (APs) in soma and dendrites of CA3 pyramidal cells and calcium imaging in the dendritic shaft and spines of CA3 pyramidal cells. I show that this algorithm allows the recording of a broad area at low speed in order to achieve a high SNR, and then pick the signal in any small compartment and resample it at high speed. This method allows preserving both the SNR and the temporal resolution of the signal, while acquiring the original images at high spatial resolution.

## Introduction

Neurons are excitable cells responsible for the conduction of electrical signals in the nervous system. For decades, electrode-based electrophysiology was the tool of choice for studying their physiological electrical activity. Its high sensitivity and temporal resolution (below 100 μs) made it a very powerful and versatile method, allowing the recording of any biological electrical event from the opening of a single channel to the discharge of a whole population of neurons (Sakmann and Neher, 1984; Neher and Sakmann, 1992). However, neurons possess complex morphologies with numerous remote and small compartments such as dendritic spines or presynaptic boutons. As electrode-based electrophysiology relies on the stable contact between a glass recording pipette (diameter of ~1 μm) and the neuron’s membrane, most studies are restricted to the recording of the cell body. Specific protocols have been designed that allow recording directly from the axon bleb, the dendritic trunk or presynaptic boutons, but sub-cellular compartments such as dendritic spines, spine neck, thin axon collaterals or en-passant boutons remain extremely difficult or even impossible to record from (Engel and Jonas, 2005; Novak et al., 2013). However it is now well accepted that small subcellular compartments can have independent electrical behavior, and that recording from the soma only reflects filtered signal coming from distinct cellular sub-compartments (Nimchinsky et al., 2002; Yasuda et al., 2004). Therefore, the main limitation of classical electrophysiology resides in its lack of spatial information. This limitation led to the development of optical methods to monitor neuronal activity.

Optical methods consist in recording the fluorescence of a reporter dye inserted into the cell. They overcome several of the limitations of electrophysiological methods described before, as they allow to record from small subcellular structures, from wide population of neurons and with use of recently developed genetically-encoded probes, even from specific cell subtypes (Scanziani and Häusser, 2009). However, compared to electrophysiological signals optical methods are limited because of the low efficiency of reporter dyes and their moderate signal to noise ratio (SNR). Neuronal signals such as action potentials (APs) or post-synaptic potentials are typically fast events with rising kinetics in the sub-millisecond range. Thus, their proper measurement needs both reporter dyes with fast kinetics and a fast detection system. However, as the acquisition speed increases, the number of photons collected decreases. Therefore, a high-speed acquisition may produce a signal with a low SNR and conversely a signal with a good SNR may require a low-speed acquisition. Moreover, increasing the acquisition speed often implies to decrease the spatial resolution of the acquired image: it is rather common to first acquire the morphology of the neuron with the full resolution of the microscope and then choose a small ROI to perform high speed line scan (see for instance Figures 3, 4 in Bialowas et al., 2015). As a result, fluorescence imaging is a constant trade-off between signal time sampling, SNR and the spatial resolution of the acquired image.

Here, I describe a method improving either SNR or time sampling of fast fluorescence signals by applying a simple shift and mean (S&M) Super-Resolution algorithm. This type of algorithms has been widely used in image computing in order to restore a high quality image from multiple low quality, blurred and/or shifted image (Elad and Feuer, 1997; Hardie et al., 1998; Shekarforoush and Chellappa, 1999; Alam et al., 2000; Elad and Hel-Or, 2001; Li et al., 2010; Létienne, 2010). To my knowledge, it has been used twice in biology by Zhao et al. (2013) and Berro and Pollard (2014), but in conditions where a low speed of acquisition was tolerated. Moreover, this technique has never been used in the field of Neuroscience. The S&M method relies on acquiring multiple recordings (or “sweeps”) of the same protocol and using the inherent jitter of the recorded signal to reconstruct the original signal at any time sampling and with a good SNR. I show practical uses of this algorithm in two different contexts: (i) as APs are among the fastest signal recorded in neurons, I first show that the S&M method allows high speed reconstruction of APs measured in voltage imaging in CA3 pyramidal cells and (ii) I then show that the S&M method also allows high resolution (HR) acquisition of calcium imaging in the dendrites and spines of CA3 pyramidal cells, therefore proving the benefits of this algorithm to reconstruct a high speed signal from a low speed but high spatial resolution image. This method greatly improves the trade-off between image resolution, SNR and speed of acquisition at the mere cost of recording a few more sweeps.

## Methods

### Preparation of Organotypic Slices

All experiments were carried out according to the European and Institutional guidelines for the care and use of laboratory animals (Council Directive 86/609/EEC and French National Research Council). Hippocampal slice cultures were prepared as follows. Briefly, postnatal day 5–7 Wistar rats were deeply anesthetized by intra-peritoneal injection of chloral hydrate, the brain removed and each hippocampus individually dissected. Hippocampal slices (350 μm) were placed on 20 mm latex membranes (Millicell) inserted into 35 mm petri dishes containing 1 ml of culture medium and maintained for up to 30 days in an incubator at 34°C, 95% O_2_–5% CO_2_. The culture medium contained (in ml) 25 MEM, 12.5 HBSS, 12.5 horse serum, 0.5 penicillin/streptomycin, 0.8 glucose (1 M), 0.1 ascorbic acid (1 mg/ml), 0.4 HEPES (1 M), 0.5 B27, 8.95 sterile H_2_O. To limit glial proliferation, 5 μM Ara-C was added to the culture medium starting at 3 DIV.

### Electrophysiological Recordings

Each slice was transferred to a submerged chamber mounted on an upright microscope equipped with a confocal system (Zeiss LSM710) and neurons were visualized using differential interference contrast microscopy through a 63× 1.0 N.A. water-immersion objective (Zeiss). Classically, CA3 pyramidal neurons were recorded in current clamp with an Axoclamp 2B by Multiclamp 700 B (Axon Instruments, Molecular Devices) and held at a voltage close to resting membrane potential (~−65 mV).

The external solution contained (mM): 125 NaCl, 26 NaHCO_3_, 3 CaCl_2_, 2.5 KCl, 2 MgCl_2_, 0.8 NaH_2_PO_4_ and 10 D-glucose, and was equilibrated with 95% O_2–_5% CO_2_. Patch pipettes (5–10 MΩ) were filled with a solution containing (in mM): K-gluconate 120; KCl 20; HEPES 10; EGTA 0.5; MgCl_2_ 2; Na_2_ATP 2; NaGTP 0.3 (pH 7.4). All recordings were made at 30°C in a temperature-controlled recording chamber (Luigs & Neumann, Ratingen, Germany).

Presynaptic APs were generated by injecting brief (10 ms) depolarizing pulses of current at a frequency of 0.1 Hz. In this condition, the AP had an inherent jitter of less than 3 ms. The voltage and current signals were low-pass filtered (3 kHz), and acquisition of 500 ms sequences was performed at 40 kHz with pClamp (Axon Instruments) version 10. Data were analyzed with a custom-made software written in Labview 10 (National Instruments).

### Voltage Imaging

CA3 pyramidal neurons were loaded with the voltage dye JPW-3028 (gift of Dr. Leslie M. Loew, University of Connecticut Health Center) in a similar protocol to Bialowas et al. (2015). Briefly, the tip of the recording pipette was pre-filled with Alexa-488 (50 μM) to visualize the morphology of the neuron and back-filled with the voltage-sensitive dye JPW-3028 (250 μM). After 5 min of whole-cell recording, Alexa diffused in the neuron and the neuronal morphology was acquired with a confocal microscope (LSM-710, Zeiss). The cell was recorded in whole-cell configuration for at least 15 min to allow extensive diffusion of the dye into the cell before the imaging protocol started. The cell was illuminated in wide field with 525 nm LED system (CoolLed, Roper Scientific) via a 63× 1.0 N.A. water-immersion objective (Zeiss). Collected fluorescence was long-pass filtered at 610 nm and projected onto a 128 * 128 pixel high speed EMCCD camera (Evolve 128, Photometrics) with a maximum frame rate of 530 Hz at full chip resolution (until 7 kHz with high cropping and binning of the image). APs were induced in the soma by injecting a 10 ms current pulse, synchronized with LED illumination. To test the algorithm in extreme sampling conditions, the camera sampling was set at 530 Hz (i.e., the fastest acquisition rate possible without binning) for 40 ms. This protocol was repeated 50 times, each sweep separated by 10 s. Electrophysiological signals recorded from the soma and CCD Frame Read-out signals were sampled and acquired at 40 kHz.

### Calcium Imaging

For calcium imaging experiments, 250 μM Oregon Green BAPTA-1 (OGB-1, Invitrogen) or 500 μM Fluo-4 (Invitrogen) were added to the pipette solution. After whole-cell access, the dye was allowed to diffuse for 10–15 min before performing experiments. The basal OGB-1 fluorescence was used to acquire the neuronal morphology by confocal stack acquisition. For high-speed imaging, the cell was illuminated in wide field with a 470 nm LED system (CoolLed, Roper Scientific) via a 63× 1.0 N.A. water-immersion objective (Zeiss). Collected fluorescence was long-pass filtered at 500 nm and projected onto an EMCCD camera (Evolve 128, Photometrics). APs were induced in the soma by injecting a 10 ms current pulse, synchronized with LED illumination and 500 Hz EMCCD acquisition for 40 ms. This protoc ol was repeated 10–15 times for OGB-1, and 70–80 times for Fluo-4, each sweep separated by 10 s. Electrophysiological signals recorded from the soma and CCD Frame Read-out signals were sampled and acquired at 40 kHz.

### Software Programming and S&M Algorithm

S&M Super-Resolution algorithm was implemented in a complete software for functional imaging. Briefly: (i) acquired images were corrected for drift movements by manual alignment; (ii) sweeps presenting no or multiple APs were discarded; (iii) remaining sweeps were aligned by AP peak or AP threshold recorded through the patch pipette; (iv) the ROI was chosen and the signal was calculated as ΔF/F; (v) the “super-sampled” signal was calculated from all available sweeps. By measuring the peak or threshold of the recorded AP in electrophysiology, every CCD Frame Read-Out were shifted to the according time on the new HR time scale. All images with the same timing in the HR time scale were added and counted, thus providing a “weight” defining the precision of each point. This created a “super-sampled” signal with a non-uniform sampling of maximum 40 kHz and a list of weights describing the precision of each point of the super-sampled signal; (vi) the super-sampled signal was resampled to the desired sampling by adding the fluorescence value of every point corresponding to the new sampling and dividing the value by the sum of their weights, therefore providing a mean of the signal; (vii) to correct for the bleaching of the fluorecent dye, a double-exponential curve was fitted using Levenberg-Marquardt least-sqaures algorithm, ignoring where the AP was localized. Then the fit was subtracted to the original data; and (viii) in the case of voltage imaging, the final result was smoothed by a weighted cubic-spline, similar to Foust et al. (2010, 2011) and Popovic et al. (2011), by minimizing the following function:

(1) $$f(x,y)=p\sum_{i=0}^{n-1}w_{i}{\left(y_{i}-f(x_{i})\right)}^{2}+(1-p)\int_{x_{0}}^{x_{n-1}}\left(f^{\prime \prime }(x\right){)}^{2}dx$$

where *p* is the balance of the smoothing factor (from 0–1, 0 meaning a linear fit and 1 no smoothing at all), *w* is the weight of the current point (thus determined by the number of images used for the averaging of this point) and *f*^″^*(x)* is the second derivative of the cubic spline function. Calcium fluorescence signals were not smoothed.

For calcium imaging, to estimate the kinetics of the signal, the obtained signal was fitted with a logistic function by the Levenberg-Marquardt least-squares algorithm.

(2) $$f(x)=\frac{A}{1+\exp^{(mu-x)*s}}$$

where *A* is the estimated amplitude of the signal, *mu* its midpoint and *s* the slope of the curve.

All of this was implemented in custom-made software developed in Labview 10, National Instruments.

## Results

### Principle of the S&M Super-Resolution Algorithm

**Figure 1:**
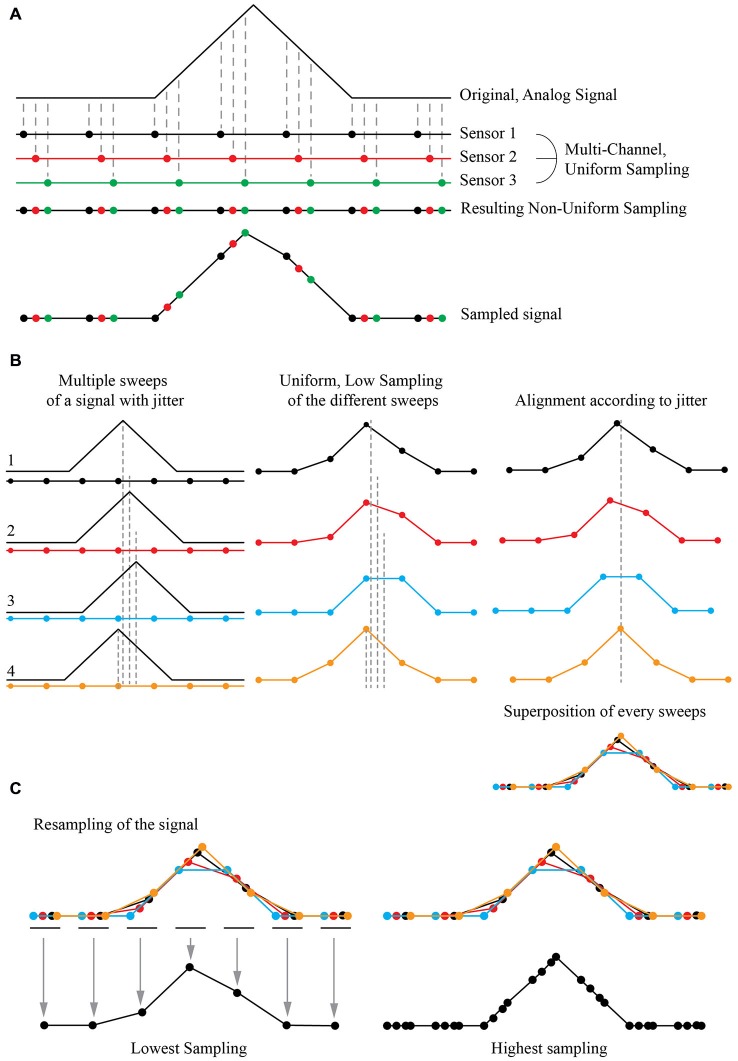
**Principle of Super-Resolution Shift and Mean (S&M) algorithm. (A)** Principle of Multi-Channel Sampling. An analog signal (top) can be sampled multiple times at low sampling rate, by a collection of three sensors with the same period but with a shift in their acquisition (middle). The final signal is a recollection of all three sensors, creating a non-uniform sampled signal (bottom). **(B)** Acquisition at low sampling and shifting of the sweeps. If the original signal has an intrinsic jitter, it is possible to reverse the situation and proceed to many acquisitions of the shifted signal with the same low sampled sensor (left). The result will give different sweeps with a low sampling (middle). If we can estimate the jitter, it is possible to align every acquisition corresponding to this jitter (right) and thus have a recollection of the data points from every sweep at the correct place compared to the original signal. This defines a high definition non-uniform sampled signal. **(C)** Resampling by averaging the data points. It is possible to choose an arbitrary sampling rate for the new signal, by simply averaging the value of each recorded points. At high resampling rate (right), some gap in the data may appear, as the final sampling is non uniform: there were not enough sweeps to fully reconstruct the signal, or the jitter of the signal was not wide enough for the reconstruction algorithm to be fully efficient.

The Shift and Mean (S&M) Super-Resolution algorithm is a typical application of multi-channel sampling thoroughly described in the literature (Papoulis, 1977). It is now widely used to increase the resolution power of numerous image sources such as blurry pictures or video acquisitions (Shmuel Peleg, 1987; Elad and Feuer, 1997; Hardie et al., 1998; Shekarforoush and Chellappa, 1999; Alam et al., 2000; Elad and Hel-Or, 2001; Li et al., 2010; Létienne, 2010). As an example, if one consider an analogic signal sampled with three sensors showing a temporal shift in their acquisition, it is possible to obtain a high resolution (HR) non-uniform sampling of the original signal by combining the three low resolution (LR) uniform sampling (Figure 1A).

If the original signal shows an inherent jitter and if one can measure it precisely, a temporal shift of the acquisition is not needed. A simple shift of the recorded signal according to this jitter produces the same signal reconstruction as in the previous case. If one can reproduce the same protocol and acquire multiple instances (or “sweeps”) of the same event with a random jitter, thus it is possible to reconstruct a “model” event at a higher non-uniform resolution by simply super-imposing all the recorded sweeps together (Figure 1B). It is then possible to resample it by simply adding all the recorded values and divide their sum by the number of images for the desired sampling, providing the mean (Figure 1C). As the reconstructed event has a high non-uniform sampling rate, the number of images used to define the new sampled point will define the “weight” of this point: if many sweeps were averaged to obtain the resampled point, it will have a bigger weight than a point resulting from one or a few sweeps to define it. By choosing the final sampling, it is then possible to choose between a low-sampling signal with many images describing each point, thus achieving a high SNR, or a high-sampling signal with a good time resolution but a low SNR.

### S&M Algorithm on a Model Case

**Figure 2:**
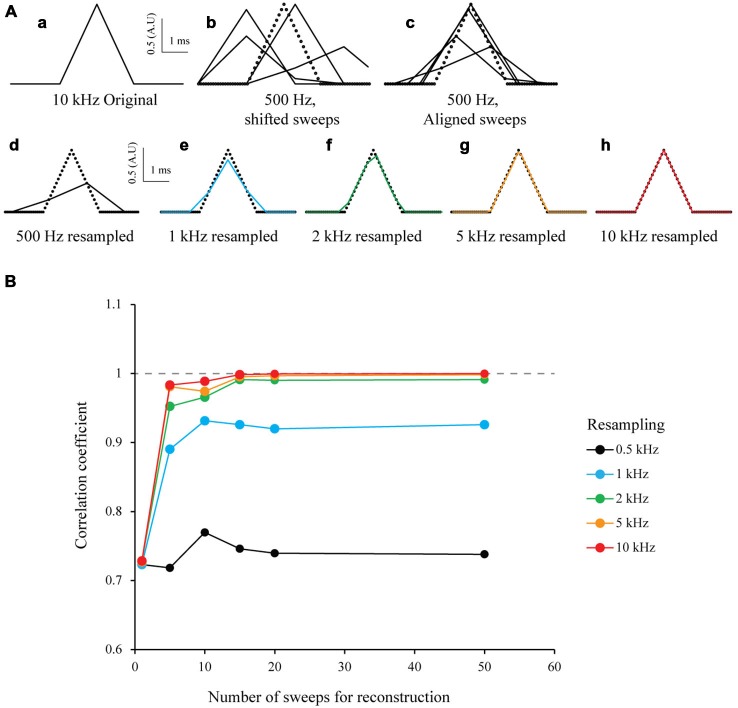
**Simulation of multi-sweep Shift and Mean algorithm for signal reconstruction. (A)** Algorithm used with model data. **(a)** 3 ms wide triangular signal created and sampled at 10 kHz. This is considered as our “ideal” High Resolution (HR) signal. **(b)** 50 Low Resolution (LR) sweeps from the signal in **(a)** obtained by first shifting it randomly in a 5 ms window and downsampling it to 500 Hz (example of four sweeps). **(C)** Low Resolution (LR) sweeps realigned according to the jitter introduced before (example of four sweeps). **(d–h)** Resampling of the 50 LR sweeps after S&M reconstruction, at 0.5, 1, 2, 5 and 10 kHz (See black, blue, green, orange and red colors, respectively). Note that at 10 kHz, the reconstructed signal fits perfectly the original one. **(B)** Impact of the number of sweeps on the resampling accuracy. We generated an increasing number of sweeps for resampling (from 1–50), and then plotted the correlation coefficient vs. the number of sweeps, for 0.5, 1, 2, 5 and 10 kHz (black, blue, green, orange and red colors, respectively). As expected, the correlation coefficient dramatically increases with the number of sweeps available and the resampling frequency.

To test the S&M algorithm, I first studied a model case in a similar way to Elad and Feuer (1997) and Elad and Hel-Or (2001). I generated an array of numbers to mimic a 3 ms-wide triangular signal of arbitrary amplitude 1 sampled at 10 kHz. This was considered as the “ideal” signal with a HR sampling (Figure 2Aa). In order to simulate many recordings (or sweeps) of this signal, I generated 50 low resolution down-sampled copies of this signal (LR sweeps) by shifting it randomly within a 5 ms window to mimic a jitter, and then down-sampled it at 500 Hz (Figure 2Ab). I then aligned every LR sweep according to the introduced jitter on an HR time scale (Figure 2Ac), added together every point with the same time and stored the number of images used for each point of the HR time scale. This procedure produces an HR image composed of the 50 shifted LR sweeps. Depending on the jitter, each point of the HR image is the result of a varying number of LR sweeps. Finally, to resample the ideal image to the desired target resolution, I added every point corresponding to the new sampling and divided the value by the sum of their weights. This produced a mean of all the LR sweeps, at desired sampling (Figure 2Ad). In an ideal case without any noise and a random jitter, if I resample it at 10 kHz I can obtain the same signal than the original HR signal, from 50 LR sweeps (Figure 2Ah, bottom right). To estimate the accuracy of the new resampled signal, I calculated the correlation coefficient *r* between the resampled ideal signal and the original ideal signal and plotted *r* vs. the number of sweeps used for resampling. As expected, the correlation coefficient increases with the resampling and the number of sweeps, similar to what has been shown by Shmuel Peleg (1987). For 50 sweeps realigned and resampled at 10 kHz, the correlation coefficient *r* is 1 (Figure 2B, red line). This clearly shows that it is possible to accurately reproduce a HR signal from multiple LR shifted copies.

**Figure 3:**
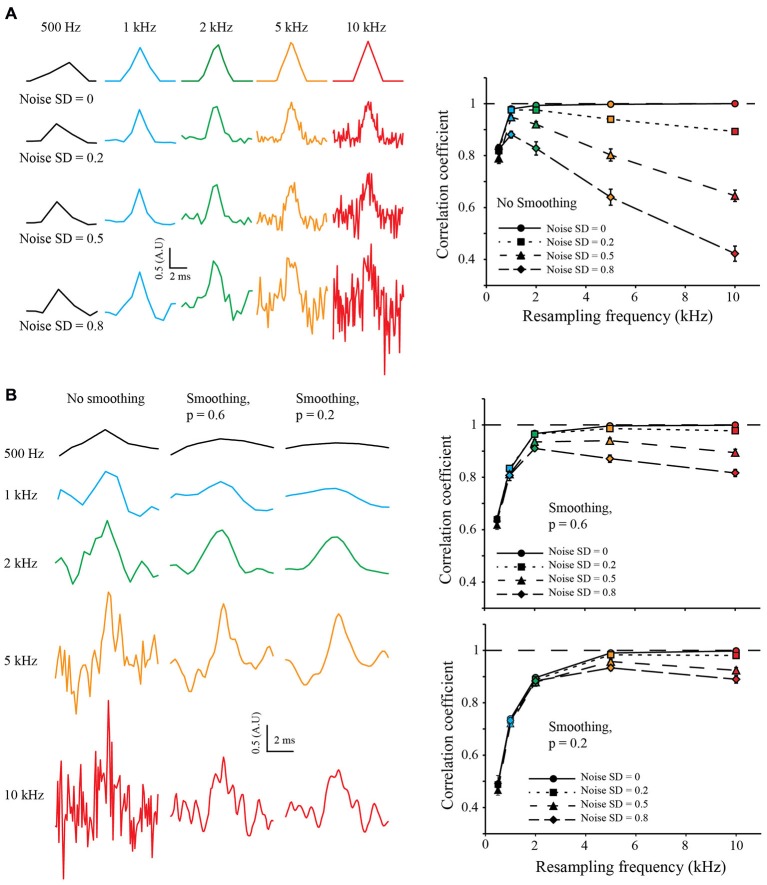
**Resampling and noise correction. (A)** Effect of noise on resampling. As previously, we generated 50 LR sweeps sampled at 500 Hz from the same 10 kHz triangular signal and then added some Gaussian White Noise with increasing standard deviation (SD) of 0.2, 0.5 and 0.8, (top, middle and bottom, respectively). This was used to simulate “good” or “bad” acquisition of the signal. We then used our algorithm to reconstruct the signal at 0.5, 1, 2, 5 and 10 kHz (Left: black, blue, green, orange and red colors, respectively) and calculated the correlation coefficient with the original HR signal (Right). In an ideal case, without any noise (*SD* = 0, plain lines), the correlation coefficient reaches one with the increase of the resampling. When adding noise, the correlation coefficient drops: as we increase the resampling, there are less and less LR sweeps to average for each point. It is interesting to note that at low resampling rate (500 Hz), the correlation coefficient is roughly the same for every noise condition: as there are many LR sweeps for each point, the averaging corrects for the noise. **(B)** Use of cubic spline smoothing for noise correction. It is possible to smooth the resampled signal with a cubic spline, in order to correct for the remaining noise. As an example, we applied two smoothing coefficients: *p* = 0.6 (soft smoothing) and *p* = 0.2 (strong smoothing; see “Methods” Section Left). When applied to low resampled signal, the smoothing decreases the correlation coefficient (right, black points). However, it improves dramatically the correlation coefficient for high resampled, noisy signals (see right, orange and red points).

To test the robustness to noise of the S&M method, I added increasing amounts of Gaussian noise to the LR copies, with a standard deviation (SD) of 0.2, 0.5 and 0.8 (meaning a SNR of 5, 2 and 1.25, respectively). For each noise level, I resampled the 50 sweeps from 500 Hz to 10 kHz. As shown on Figure 3A (left), the noise increases with the resampling, as the number of LR copies used to calculate each point of the resampled signal decreases. Accordingly, the correlation coefficient increases at first with the resampling, but then drops quickly, due to the increase in noise (Figure 3A, right).

In order to correct for noise variations remaining from the mean of the copies, I used a weighted cubic-spline smoothing (Foust et al., 2010; Popovic et al., 2011). As the weight of each point is known, and thus the precision describing them, it is possible to fine-tune the cubic spline smoothing (the smoothing applied to each point is inversely related to the number of LR copies used to calculate it). I used two values for the smoothing coefficient *p* of the cubic spline equation (see “Methods” Section): 0.6 and 0.2, equivalent to fine and coarse smoothing, respectively (Figure 3B, left). It is interesting to note that smoothing tends to decrease the correlation coefficient when applied to LR resampling but increases it for HR resampling (Figure 3B, right). This shows that it is possible to choose a HR resampling and then correct the remaining noise with weighted cubic spline smoothing.

I decided to try our S&M algorithm on two different paradigms where acquisition speed and SNR are particularly critical: voltage imaging and calcium imaging on CA3 pyramidal cells.

### S&M Algorithm Applied to Voltage Imaging

Voltage imaging is a pioneering technique, which allows recording voltage shifts by using voltage-sensitive fluorescent dyes (Canepari et al., 2008; Foust et al., 2010, 2011; Holthoff et al., 2010; Popovic et al., 2011, 2015a,b; Bialowas et al., 2015). However, voltage dyes have still a low efficiency, which means that a good SNR is hard to obtain. Moreover, electrophysiological signals like APs are extremely fast and need a high acquisition speed. Thus, recording neuronal activity with voltage-sensitive dyes is ideally suited for S&M Super-Resolution: (i) the electrophysiological signal recorded via the patch pipette has a high sampling rate and a high SNR, but is limited to the soma or large neuronal structures; (ii) the fluorescence signal acquired via voltage dyes has a low sampling rate and a low SNR but can be recorded in really small structures, like thin dendrites or dendritic spines; and (iii) APs triggered by current steps have an inherent jitter and we can measure it precisely using electrophysiological measurements. This means that combining voltage imaging and electrophysiology recording should allow us to use the S&M algorithm and thus reconstruct the fluorescent signal at a higher sampling rate than the original one.

**Figure 4:**
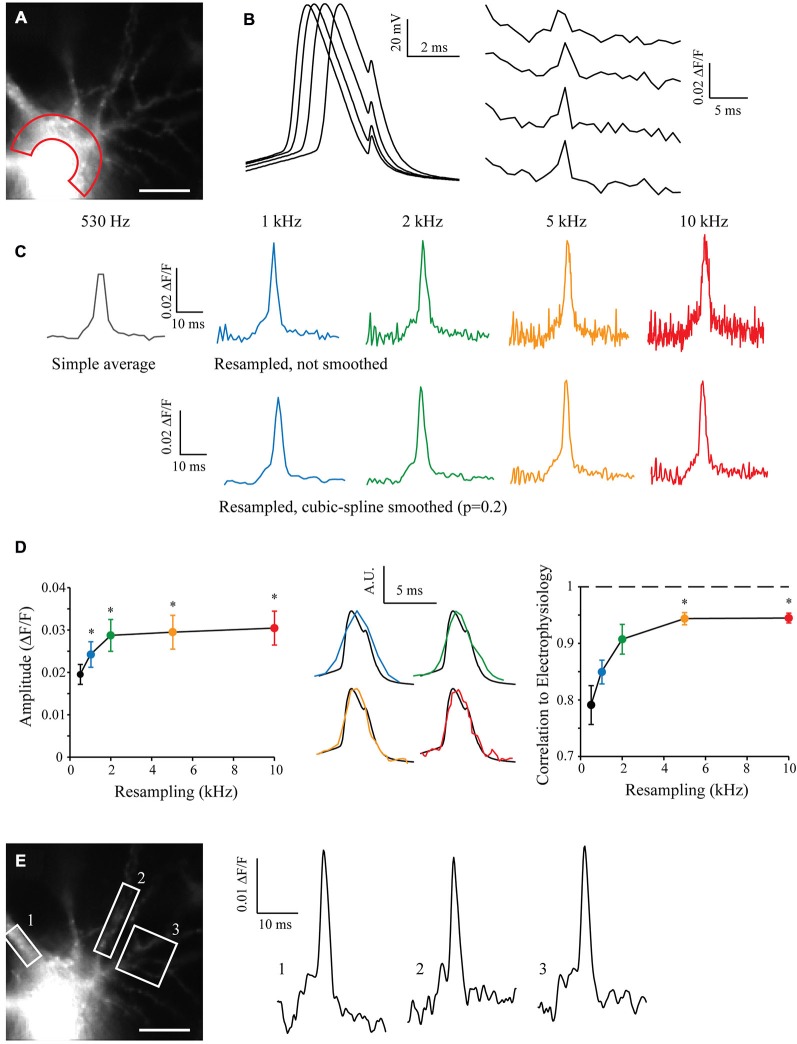
**Shift and Mean algorithm with voltage imaging. (A)** Experimental setup showing a CA3 pyramidal neuron filled with Alexa 488 and JPW3028, visualized at HR with the 530 Hz EMCCD camera. Scale Bar: 10 μm. Red area: Region of Interest (ROI) used for acquisition of the voltage imaging. **(B)** Voltage signals acquired at the soma in electrophysiology (left) and in the ROIs with voltage imaging (right). Note the inherent jitter of each Action potentials (APs) recorded in electrophysiology and the really low sampling of the APs recorded in voltage-imaging. **(C)** Example of reconstructed signal from 50 Low Sampling acquisitions, at 530 Hz (original sampling of the EMCCD camera, equivalent to a simple average), 1, 2, 5 and 10 kHz (black, blue, green, orange and red traces, respectively). Top: without cubic spline smoothing. Bottom: with cubic spline smoothing (*p* = 0.2). Note that the AP becomes taller and sharper with the increase in the resampling, but the noise increases as well. **(D)** Statistics showing the evolution of the amplitude (left) and shape of the reconstructed APs according to the resampling. Note the dramatic improvement of the shape of the AP acquired in imaging when increasing the resampling (middle and right, 1, 2, 5 and 10 kHz showed in blue, green, orange and red traces, respectively) vs. the AP acquired in electrophysiology (black; *n* = 7). Stars: Wilcoxon test vs. the original sampling (black dot), *p* < 0.05. **(E)** As the acquired original images have a HR, it is possible to measure the voltage signal at many different points such as in ROI 1, 2 or 3 at the same time and for any resampling. Examples are shown at 5 kHz, and smoothed (*p* = 0.2).

I filled a CA3 pyramidal neuron with Alexa488 and JPW3028 in order to reveal neuronal morphology and acquire voltage-dependent fluorescence when triggering an AP. After acquisition of 50 sweeps with the CCD camera at 530 Hz (the maximum sampling rate possible with our camera without binning), I measured the JPW3028 fluorescence in the soma of recorded cell (Figure 4A, and see “Methods” Section). The electrophysiological recordings from the soma showed a typical jitter of the AP in each sweep (Figure 4B, left). At a sampling rate of 530 Hz the fluorescence signal is far from accurate (Figure 4B, right). I resampled the signal from 530 Hz to 10 kHz, with or without a cubic spline smoothing (*p* = 0.2; Figure 4C). Interestingly, increasing the resampling made the AP significantly taller (Figure 4D, left). When compared to the AP recorded in electrophysiology at 10 kHz in the same cell, the correlation coefficient grew from 0.8 to 0.95 with the increase in resampling (Figure 4D, middle and right). This demonstrates an improvement in the kinetics of the measured signal, compared to a low resampling.

The original sweeps were acquired with the maximum resolution of the CCD camera without binning or cropping of the sensor. This preserved the spatial resolution of the images and thus it is possible to analyze the fluorescence signal in many other different compartments in the cell, such as the soma or different dendrites. By choosing regions of interest (ROIs) on proximal dendrites and using the S&M super-resolution algorithm, it is possible to show that the AP can be accurately measured in these subcellular compartments (Figure 4E).

### S&M Algorithm Applied to Oregon Green BAPTA-1 Imaging

**Figure 5:**
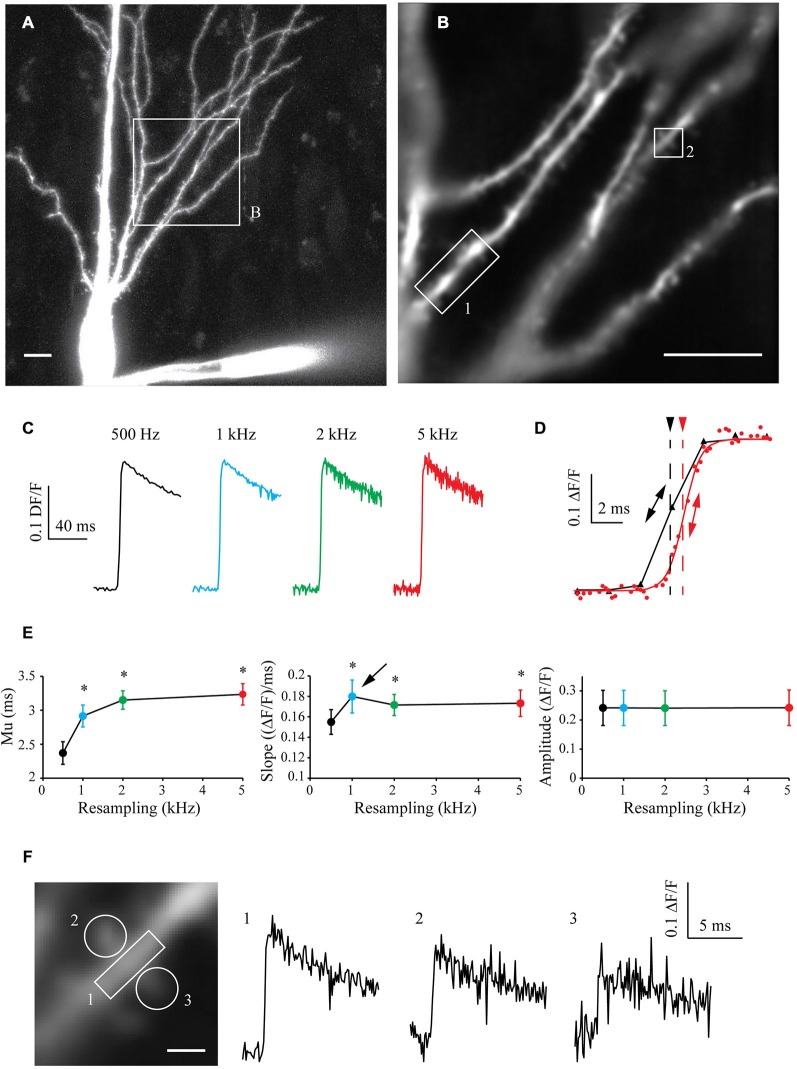
**Shift and Mean algorithm with OGB-1 calcium dye. (A)** Confocal visualization of a CA3 pyramidal neuron filled with Oregon Green BAPTA-1. Scale Bar: 10 μm. **(B)** ROI used for CCD acquisition. **(B)** Example of CCD acquisition of the dendritic tree showed in **(A)**. Scale Bar: 5 μm. **(C)** Examples of calcium fluorescence acquired from ROI 1 and resampled at 0.5, 1, 2 and 5 kHz (black, blue, green and red traces, respectively). Note that the noise increases with the sampling rate, as there are less and less images to describe each points. As the acquired signal is slow, the amplitude remains constant. **(D)** Enlargement of the same data resampled at 0.5 and 5 kHz (black and red traces, respectively) showing the improvement in the accuracy of the kinetics of the signal. The midpoint of the rising phase is shifted to the right (simple arrows) and the slope is steeper (double arrows) when increasing the resampling. **(E)** Quantitative data showing the evolution of the midpoint of the rising phase (mu), the slope and the amplitude of calcium signals recorded in seven cells. Note that the midpoint is shifted and the slope is increased when increasing the resampling of the acquired signal. However, the slope presents a maximum at 1 kHz resampling (arrow), then a slight decrease. This shows that kinetics first improves with the resampling, and then are blurred by the noise increase. Stars: Wilcoxon test vs. original sampling (black dot), *p* < 0.05. **(F)** Left: Enlargement of the ROI 2 showed in **(B)**. Right: calcium events recorded in ROIs 1, 2 and 3, resampled at 1 kHz.

To test the algorithm on another paradigm, I filled CA3 pyramidal cells with 250 μM High-affinity Oregon Green BAPTA-1 [OGB-1, Kd ~170–200 nM (Sabatini et al., 2002; Paredes et al., 2008)]. After 10 min of dye filling, the neuronal morphology was acquired with a LSM-710 confocal microscope (Figure 5A) and a cell sub-area was chosen for calcium imaging (Figure 5B). I recorded 10–15 sweeps of 1 s with an illumination of 160 ms synchronized with an AP triggered through the patch pipette and CCD acquisition at 500 Hz. The OGB-1 fluorescence signal acquired in one ROI at different resampling frequencies is shown in Figure 5C. When increasing the resampling frequency, the SNR decreases due to the decrease in the number of images used for averaging. Interestingly however, the kinetics of the rising phase of the calcium signal are significantly improved with the resampling (Figure 5D). When increasing the resampling, the midpoint mu is continuously shifted and the slope shows first an increase for 1 kHz resampling and then a slight decrease of its value for 2 and 5 kHz resampling. This reveals first an improvement of the kinetics until 1 kHz, which is then dampened by the increase in noise at high resampling frequencies (see Figure 5D and statistics in Figure 5E). This shows that resampling significantly improves the definition of the kinetics of the measured signal, and that 1 kHz resampling is the optimal choice for the number of acquisitions performed (10–15 recorded sweeps).

In a similar way to what was presented in Figure 4E, acquiring the fluorescence of the whole area at low speed allowed to record a large area of the neuron with a good SNR. In the same experiment, I could therefore analyze the fluorescence signal in another ROI (ROI2, Figure 5F, left) and then resample the signal recorded in this dendritic shaft (Figure 5F, right, trace 1) or nearby spines (Figure 5F, right, traces 2 and 3). This example demonstrates that large areas of interest can be monitored and recorded at low speed to obtain a good SNR, and that the signal can be reconstructed at high sampling even in small subcellular compartment such as dendritic spines.

### S&M Algorithm Applied to Fluo-4 Calcium Imaging

**Figure 6:**
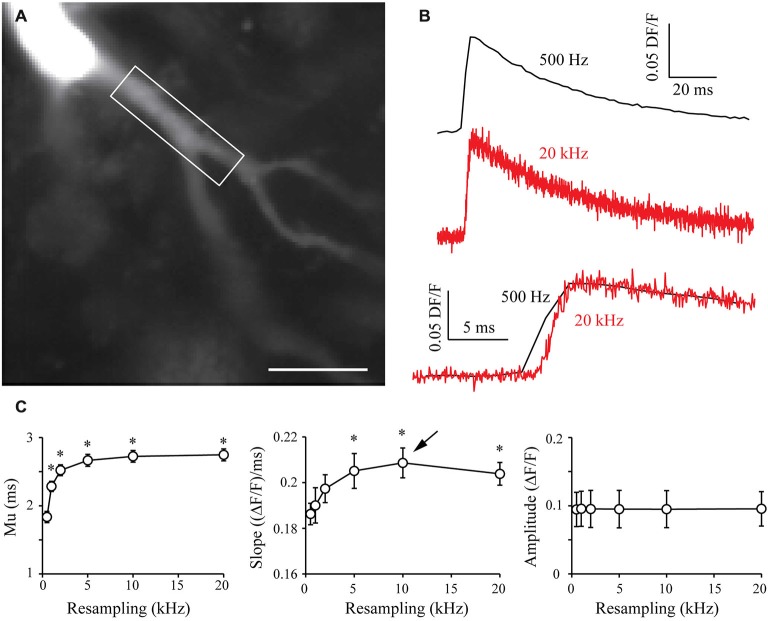
**Shift and Mean algorithm with Fluo-4 calcium dye. (A)** CCD acquisition of a CA3 pyramidal neuron filled with 500 μm Fluo-4. Scale Bar: 20 μm. Rectangle: ROI used for signal resampling. **(B)** Examples of calcium signals acquired at 530 Hz (top, black) and resampled at 20 kHz (middle, red). Bottom: Comparison of the kinetics of the two signals. **(C)** Quantitative data showing the evolution of the midpoint of the rising phase (mu), the slope and the amplitude of calcium signals recorded in seven cells. As in OGB-1 experiments, the midpoint is shifted and the slope is increased when increasing the resampling of the acquired signal. As I recorded more sweeps (70–80), the slope presents a maximum at 10 kHz resampling (arrow). This shows that it is possible to resample a signal at high sampling when acquiring a large number of sweeps. Stars: Wilcoxon test vs. original sampling (500 Hz), *p* < 0.05.

In order to generalize the benefits of the S&M algorithm, I also performed calcium imaging experiments with the Fluo-4 calcium dye, which displays different kinetics from Oregon Green BAPTA-1. Fluo-4 has a Kd of around 345 nM, making it a faster calcium reporter than OGB-1 (Kao and Tsien, 1988). In a similar way to what was presented in Figure 5, I filled CA3 pyramidal neurons with 500 μm Fluo-4, waited 10 min for dye filling and recorded 70–80 sweeps of 1 s with an illumination of 160 ms synchronized with an AP triggered through the patch pipette and CCD acquisition at 500 Hz (Figure 6A). Recording such a large number of sweeps allows resampling at high frequencies, which might be necessary to accurately monitor the faster kinetics of Fluo-4. Indeed, Figure 6B shows that 20 kHz resampling yields much more accurate kinetics than the 500 Hz averaged signal. When plotted against resampling frequency, the midpoint mu of the rising phase shows the same behavior as with OGB1-dye, and is exponentially improved with increasing resampling frequencies (Figure 6C, left). The slope of the Fluo-4 signal is steeper than the slope of OGB-1 signals, increases until 10 kHz resampling and shows a slight decrease for 20 kHz (Figure 6C, middle). This directly shows that S&M algorithm can be applied to dyes with diverse kinetics, and that faster dyes only require to record a larger number of sweeps: acquiring 70–80 sweeps was sufficient to perform 10 KHz resampling and reveal the faster Fluo-4 kinetics.

## Discussion

In this paper, I present a way to use an algorithm previously used in image processing in functional imaging of living neurons. By recording multiple sweeps of a same protocol and measuring the precise jitter of each event, it is possible to reconstruct a signal with a higher sampling frequency than the original sweeps and increase the SNR as well. To my knowledge, two previous studies rediscovered this algorithm in their work (Zhao et al., 2013; Berro and Pollard, 2014) and named it “Event-Correlation Microscopy” (ECOM) or “Temporal Super-Resolution”, respectively. It is originally called Shift and Add or Shift and Mean Super Resolution (Elad and Hel-Or, 2001) and I show here that it can be of great interest for imaging fast signals in living neurons.

### Requirements

The main requirement comes from the need to perfectly synchronize fluorescence acquisitions between each other. Electrophysiology and fluorescence imaging are one of the methodological combinations suitable for this algorithm, as electrophysiology has a high acquisition sampling rate and thus allows a very precise alignment of the acquired fluorescence signal.

### Limitations

The first limitation for this algorithm comes from the idea that every recorded sweep is equivalent to any other. In this case for instance, if the AP velocity was varying through recorded sweeps I would not be able to reconstruct it properly in voltage imaging. Moreover, when resampling the signal from many different sweeps, we lose information about the trial-to-trial variability of the signal, and this variability could contain precious information. However, if the original acquisition frequency is chosen carefully, one can analyze every recorded sweep to measure the variability of the signal and then resample it using the S&M algorithm, in order to preserve all the information. For instance, in our calcium imaging reconstruction, it is possible to measure the variation of the amplitude of each single event and then analyze the kinetics of the resampled model event.

A second limitation comes from the principle of multi-channel sampling: when increasing the number of sweeps, the maximum sampling obtained will be roughly the original sampling multiplied by the number of sweeps recorded. As an example with our calcium imaging experiments, the maximal sampling rate will be around 500 Hz * 10 *sweeps* = 5 kHz. However, our experiments showed that the kinetics degrade before this theoretical limit. This is probably due to the fitting of the sigmoid curve used to estimate the different parameters. As noise increases with resampling, the accuracy of the fit decreases.

The third limitation comes from the need to precisely synchronize each fluorescence acquisition with each other. This means it will be difficult to use it for spontaneous events, or these spontaneous events have to be detected by another method and then re-aligned in order to resample the imaging signal.

The last limitation is the duration needed to record many sweeps of the same protocol, and therefore the quality of the recording throughout the entire experiment. As we increase the number of sweeps, the duration of the protocol increases as well and extra care has to be taken to preserve the recording conditions, otherwise the signal recorded during the last sweeps of the experiment will significantly differ from the signal recorded during the first ones. This can be overcome by recording each sweep for a really short time, thus limiting illumination and photo-damage, and by regularly monitoring the recording conditions of the cell. As an example, the voltage imaging experiments lasted approximately 10 min (50 sweeps ^*^ 10 s *intersweeps* = 500 s), but the calcium imaging experiments were much shorter, lasting approximately 3 min (15 sweeps ^*^ 10 s *intersweeps* = 150 s). Light illumination and fluorescence recordings were kept very short: 40 ms during each sweep for voltage imaging, and 160 ms during each sweep for calcium imaging.

### Advantages

The main advantage of this technique is the great flexibility between the sampling of the recorded signal, the final SNR and the spatial resolution of the original images acquired. As this method allows keeping a correct spatial resolution without sacrificing the final temporal resolution, one can record a signal in a very large area at low speed and finally resample the signal in small compartments at high speed, in a single experiment. This makes this tool extremely useful for population recordings, as long as one can record many sweeps of the same experiment.

For example in the calcium imaging experiments, I was able to record the calcium signal in a large area of the dendritic tree and to visualize the results with either a high SNR or a high sampling, from the same experiment. As the original images have a good spatial resolution, I was able to measure the calcium signal in dendrites and spines as well, at any sampling rate and in the same experiment.

### Potential Applications

This great flexibility allows extending imaging protocols far above the limitations of classical CCD cameras, such as the one used in the current study: acquisition speed is not an issue anymore. For instance, it is interesting to note that, in the case of the Fluo-4 calcium imaging experiments, the acquisition of 70–80 sweeps at 500 Hz allowed to reach higher sampling frequencies than previous studies using fast scanning RAMP microscopy (Otsu et al., 2014).

I showed here two examples of calcium imaging using two slightly different high-affinity calcium dyes, OGB-1 and Fluo-4. Although high affinity dyes present rather slow kinetics (Kao and Tsien, 1988; Canepari and Mammano, 1999) and thus are not perfectly suited for high-speed imaging, these experiments demonstrate that it is possible to reconstruct the signal generated by these dyes at high sampling frequency, and that S&M high speed resampling of acquired fluorescence might prove particularly useful for imaging fast calcium events. With the use of the fast kinetics calcium dye Oregon Green BAPTA-5N (OG5N, Kd ~35–46 μm), Jaafari et al. (2014) recorded calcium signal at 20 kHz in CA1 pyramidal cells. In order to reach such high acquisition speed, they had to increase the binning of their CCD camera to strips of 26 * 4 pixels and they averaged 16–64 trials to obtain a satisfying SNR. The S&M algorithm may have been an alternative tool to reach such high sampling without sacrificing as much of the spatial resolution.

This flexibility can be extended to any kind of functional fluorescence imaging. For instance, many pioneering works detailed AP propagation in neurons using voltage-sensitive dyes and confocal or two-photon microscopy (Palmer and Stuart, 2009; Acker et al., 2011; Acker and Loew, 2013). However to do so, the authors had to compromise their spatial resolution by using high-speed line scans or even point scans and to average many trials in order to obtain a good SNR. The S&M algorithm would have been of great use in this case, as the gain in acquisition speed does not necessarily come at the price of poor image resolution. The S&M algorithm would also be especially useful when using two-photon microscopy, as fluorophore excitation efficiency increases with the square of the exposure time (Denk et al., 1990).

To extend this further, if one uses a fast 2-photon or confocal microscope it is theoretically possible to acquire full z-stacks of a neuron synchronized with triggered APs, and then visualize the propagation of the AP in the full dendritic arborization. After acquisition of multiple sweeps, the S&M algorithm allows to choose between SNR, spatial resolution and final sampling of the observed signal.

## Author Contiributions

SR conceived the project, performed the experiments and wrote the paper.

## Funding

Supported by *Institut National de la Santé et de la Recherche Médicale*, *Centre National de la Recherche Scientifique*, *Agence Nationale de la Recherche* (ANR Blanc “REPREK” – 11-BSV4-016-01 and ANR Santé and Bien-Etre “AXODE” – 14-CE13-0003-02).

## Conflict of Interest Statement

The author declares that the research was conducted in the absence of any commercial or financial relationships that could be construed as a potential conflict of interest.
